# A Residual Dense Attention Generative Adversarial Network for Microscopic Image Super-Resolution

**DOI:** 10.3390/s24113560

**Published:** 2024-05-31

**Authors:** Sanya Liu, Xiao Weng, Xingen Gao, Xiaoxin Xu, Lin Zhou

**Affiliations:** 1 Xiamen Key Laboratory of Mobile Multimedia Communications, College of Information Science and Engineering, Huaqiao University, Xiamen 361021, China; liusy@hqu.edu.cn (S.L.); 22014082024@stu.hqu.edu.cn (X.W.); 2 School of Opto-Electronic and Communication Engineering, Xiamen University of Technology, Xiamen 361024, China; gaoxingen@xmut.edu.cn; 3 Institute of Microelectronics Chinese Academy of Sciences, Beijing 100029, China; xuxiaoxin@ime.ac.cn

**Keywords:** RDAGAN, single image super-resolution, microscopic image, generative adversarial network, image processing

## Abstract

With the development of deep learning, the Super-Resolution (SR) reconstruction of microscopic images has improved significantly. However, the scarcity of microscopic images for training, the underutilization of hierarchical features in original Low-Resolution (LR) images, and the high-frequency noise unrelated with the image structure generated during the reconstruction process are still challenges in the Single Image Super-Resolution (SISR) field. Faced with these issues, we first collected sufficient microscopic images through Motic, a company engaged in the design and production of optical and digital microscopes, to establish a dataset. Secondly, we proposed a Residual Dense Attention Generative Adversarial Network (RDAGAN). The network comprises a generator, an image discriminator, and a feature discriminator. The generator includes a Residual Dense Block (RDB) and a Convolutional Block Attention Module (CBAM), focusing on extracting the hierarchical features of the original LR image. Simultaneously, the added feature discriminator enables the network to generate high-frequency features pertinent to the image’s structure. Finally, we conducted experimental analysis and compared our model with six classic models. Compared with the best model, our model improved PSNR and SSIM by about 1.5 dB and 0.2, respectively.

## 1. Introduction

Regardless of the magnification and numerical aperture of the objective lens used, the imaging throughput of current microscopes is typically only in the order of ten megapixels [1]. This leads to a compromise between high resolution and Field of View (FOV) when imaging. However, in biomedical research such as histopathology, hematology, and neuroscience, there is a growing need for high-resolution imaging of large samples. In order to accurately resolve cellular-level life activities at the scale of the entire sample, a balance between the global structure and microscale local details is required, along with quantitative analysis. Faced with this challenge, Super-Resolution (SR) imaging techniques were developed. Single Image Super-Resolution (SISR) reconstruction [2] aims to reconstruct a High-Resolution (HR) image from an input Low-Resolution (LR) image.This technique is widely used in critical fields, including bright field micrographs [3], fluorescent imaging [4,5], remote sensing images [6,7], and surveillance videos [8]. Traditional SISR reconstruction algorithms can be divided into three categories: interpolation-based [9,10], reconstruction-based [11,12], and learning-based [13]. Interpolation-based algorithms have the advantages of simple principle and easy implementation. However, these methods have difficulty recovering the detailed information of the image, and the reconstructed image is seriously distorted. Reconstruction-based algorithms first generate the constraints for SR reconstruction according to the imaging process of LR images, establish the corresponding mathematical model, and finally reconstruct HR images. However, when the magnification is high, it is still difficult to recover enough high-frequency information. The learning-based reconstruction algorithm mainly establishes the mapping relationship between LR and HR hyperspectral images, and then reconstructs according to the mapping relationship. In recent years, with the rapid development of deep learning, the excellent learning ability of convolutional neural networks also brings new opportunities for the development of SR imaging technology.

Specifically, Dong et al. [14] used deep learning in image SR reconstruction by introducing Convolutional Neural Networks (CNNs). This groundbreaking work paved the way for subsequent innovations. Kim et al. [15] introduced the Very Deep Super Resolution (VDSR) model, which leverages a deeply constructed neural network combined with residual learning for image reconstruction. This approach effectively addresses the issue of image size reduction caused by successive convolutions. Lai et al. [16] proposed the Laplacian Pyramid Super-Resolution Network (LapSRN) model, which employs a Laplacian pyramid to progressively reconstruct the image and perform feature extraction through a residual structure. Ledig et al. [17] advanced the field by incorporating residual blocks within the structure of a Generative Adversarial Network (GAN) to create the Super-Resolution Generative Adversarial Network (SRGAN) model. The use of GANs significantly improves the visual perception quality of the generated images, making them more closely resemble real images. Lim et al. [18] developed the Enhanced Deep Super-Resolution (EDSR) model, which refines the residual structure found in the SRGAN model for improved performance. Zhang et al. [19] contributed by introducing the Residual Dense Block (RDB) structure and the Residual Dense Network (RDN) model, further advancing the capabilities of SR reconstruction.

In terms of microscopic image reconstruction, Zhang et al. [20] proposed Registration-Free GAN Microscopy (RFGANM) workflow by combining an SRGAN network with an optical microscope and degradation model to achieve deep learning SR in large FOV, and improve the resolution of wide-field microscopy and light-sheet fluorescence microscopy images. Wang et al. [21] used a GAN network to develop SR techniques for cross-modal fluorescence microimaging by simulating the mapping relationships between different imaging techniques, such as wide-field fluorescence imaging, confocal to STED, and TIRF to TIRF-SIM, through deep learning. Van Sloun [22] applied deep learning to ultrasound microscopy SR study and proposed the Deep-ULM model, which is based on the U-Net network to reduce the effect of diffraction and obtain HR images in real time. Li et al. [23] achieved high-quality reconstruction of ordinary wide-field fluorescence images to SIM SR imaging results using a Deep Fourier Channel Attention (DCFA) network, greatly contributing to the development of Super-Resolution in microscopic images.

It can be seen that, although great progress has been made in the SR of microscopic images, most of them are focused on fluorescence images in a special way. We know that the features of fluorescence microscopy images are significantly different from those of cell images. Additionally, despite the above SR methods having achieved good results, there are some shortcomings in any of them. These include the absence of specialized datasets for microscopic images, which hampers model training; the inadequate use of hierarchical features in LR images by GAN-based SR models; insufficient utilization of information across convolutional layers due to varying receptive fields; a lack of prioritization in reconstructing feature map information, leading to a suboptimal focus on critical details; and the reliance on pixel-level reconstruction errors as loss functions, which fails to capture high-frequency details and often results in overly smooth and potentially inaccurate images.

In order to respond to these challenges, this paper has carried out corresponding optimization work on cell microscopy images. The specific contributions are described as follows:

(1) We produce a dataset of high- and low-resolution images of four cell types using microscope acquisition. You can find the dataset at https://github.com/wxsssss/RDAGAN/tree/main (accessed on 15 January 2024).
(2) In order to fully utilize the hierarchical features of the original image, we propose a Residual Dense Attention Generative Adversarial Network (RDAGAN), whose generator uses RDABs with increased attention mechanisms.
(3) To reconstruct the high-frequency features associated with HR images, we add a feature discriminator to the original discriminator and optimize the loss function.
(4) Our proposed optimized model is compared with six classic models. Compared to the best performing model among them, PSNR and SSIM have improved by 1.5 dB and 0.2, respectively.

## 2. Proposed Methodology

Our model is based on the aforementioned RFGANM [20] that has been used for microscopic cell image SR, constructing a generator network architecture composed of four main sections: the feature extraction part, the residual dense attention part, the dense feature fusion part, and the reconstruction part. The input LR image first passes through the feature extraction section, where initial features are extracted. These features are then processed by the residual dense attention section. Here, the RDB extracts rich local features through densely connected convolutional layers, fully leveraging the hierarchical features of the layers. The local feature fusion within the RDB allows for an adaptive and more effective integration of previous and current local features, thus stabilizing the training of broader network. An attention mechanism is added after each RDB, first applying a channel attention module to obtain a weighted result, followed by a spatial attention module to refine the weighting. This enables the network to concentrate on information that is most beneficial for image reconstruction. The RDB and the attention module together constitute the Residual Dense Attention Block (RDAB). After extracting dense local features through a series of residual dense attention modules, dense feature fusion is proposed to mine multi-level features from a global perspective. Additionally, a feature discriminator [24] is introduced to differentiate the detailed features of SR and HR images, encouraging the reconstructed image to generate more high-frequency features instead of noise.

### 2.1. Microscopic Cell Image Dataset

We used a microscope to capture cell images using both 40× and 20× objective lenses simultaneously to establish our dataset. We use the EasyScan NFC 300 microscope from Motic, which is a high-performance fully automatic digital scanning microscope equipped with advanced optical and imaging technology, to provide high-quality microscopic images. The microscope features fast and precise autofocus capabilities to ensure image clarity throughout the scanning process. It employs a high-quality infinity-corrected optical system that delivers clear and distortion-free images. The optical system incorporates advanced correction design to minimize common optical aberrations such as spherical aberration, chromatic aberration, and coma, providing accurate imaging quality. Additionally, the system undergoes rigorous color correction and transmission curve optimization to ensure uniform light transmission across different wavelengths, reducing color distortion and light loss. We perform center cropping on the captured images. HR images were cropped to a resolution of 1024 × 1024 pixels, while LR images were cropped to 512 × 512 pixels, reflecting the 2× objective relationship. In constructing the dataset, we faced challenges in precisely matching HR and LR images. To address this, we employed Bicubic Downsampling (BD) to generate corresponding LR images from their HR counterparts. These LR images were then used for both reconstruction purposes and performance comparison. Finally, we created 800 pairs of images for training data and 200 pairs of image val data for reconstruction for each of four cell images. You can refer to Appendix A for relevant characteristics of microscopic cell images.

### 2.2. Residual Dense Blocks for Fusion CBAM

**Figure 1:**
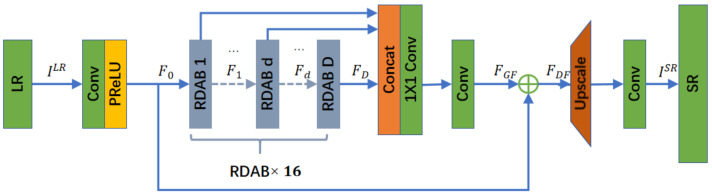
Residual Dense Attention Generative Adversarial Network (RDAGAN) framework.

The structure of our generator network is shown in Figure 1, consisting of four parts: a shallow feature extraction network, a RDAB, a Dense Feature Fusion (DFF), and an upsampling network. The input and output of the generator network are represented by $I_{LR}$ and, respectively, $I_{SR}$. A convolutional layer is used to extract shallow features $F_{0}$ of the LR image and perform global residual learning, while $F_{0}$ also serves as an input to the RDAB. $F_{0}$ denotes the convolution operation on the LR image. Assuming that there are D RDABs in the generator, the Dth RDAB can be represented as follows:

(1) $$\begin{array}{cc} F_{d} & =H_{RDAB,d}\left(F_{d-1}\right)=H_{RDAB,d}\left(H_{RDAB.d-1}\left(\cdots \left(H_{RDAB,1}\left(F_{0}\right)\right)\cdots \right)\right) \end{array}$$

where $H_{RDAB,d}$ represents the operation of the dth RDAB, which is a composite function of convolution and ReLU operations. Meanwhile, $F_{d}$ is generated by the dth RDAB fully utilizing the features from each convolutional layer within the block and $F_{D}$ can be considered as a local feature. The generator RDABs in this paper are 16 in total.

After extracting the hierarchical features with a set of D RDABs, further DFF is performed, including Global Feature Fusion (GFF) and Global Residual Learning (GRL). DFF fully utilizes the features of all previous layers; $F_{DF}$ is the output feature mapping of the composite function $H_{DFF}$ of the DFF module, which can be expressed as follows:

(2) $$F_{DF}=H_{DFF}\left(F_{0},F_{1},\cdots F_{D}\right)$$

Local and global features are extracted and fused in the LR space to obtain $F_{DF}$, which is used as an input to the upsampling network. The upsampling network used in this invention is the same as SRGAN. The entire network of the generator as a whole can be represented as follows:

(3) $$I_{SR}=H_{Generator}\left(I_{LR}\right)$$

**Figure 2:**
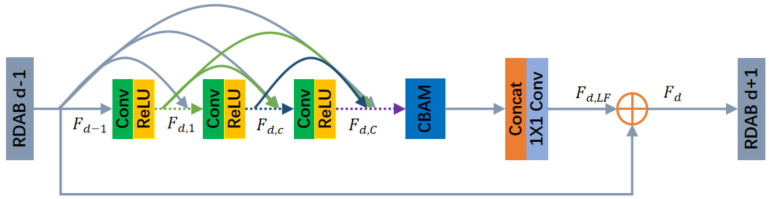
Residual Dense Attention Blocks (RDABs).

The structure of the RDAB is shown below in Figure 2. Our RDAB contains a dense connectivity layer, local feature fusion (LFF), Local Residual Learning (LRL), and CBAM, thus forming a Continuous Memory (CM) mechanism.

Denote the input and output of the dth RDAB by $F_{d-1}$ and $F_{d}$, respectively. The outputs of the previous RDAB and each convolutional layer of that RDAB are directly connected to all subsequent layers, which not only preserves the feed-forward properties but also extracts the local dense features. $F_{d,c}$ denotes the output of the cth convolutional layer in that RDAB, and $F_{d,C}$ denotes the output of the last convolutional layer of the densely connected layers in that RDAB. The purpose of LRL is to further improve the information flow, and the final output of the dth RDAB is denoted as follows:

(4) $$F_{d,LF}=H_{LFF}^{d}\left(\left[F_{d-1},F_{d},\cdots ,F_{d,c},\cdots F_{d,C}\right]\right)$$

(5) $$F_{d}=F_{d-1}+F_{d,LF}$$

where $H_{LFF}^{d}\left(\cdot \right)$ denotes the fusion of all layer feature information in the dth RDAB. DFF is feature fusion and residual learning for each RDAB feature obtained to utilize hierarchical features in a global manner. DFF includes GFF and GRL. The global features are extracted by fusing the features of all RDABs.

(6) $$F_{GF}=H_{GFF\left(\left[F_{1},\cdots ,F_{D}\right]\right)}$$

(7) $$F_{DF}=F_{0}+F_{GF}$$

The RDAB introduces a continuous memory mechanism that allows previous RDABs to directly access each layer of the current RDAB. By using LFF, it is possible to train the deep network stably, while LRL can further optimize the information flow and gradient. In addition the RDAB uses GFF and GRL to further extract global features.

**Figure 3:**
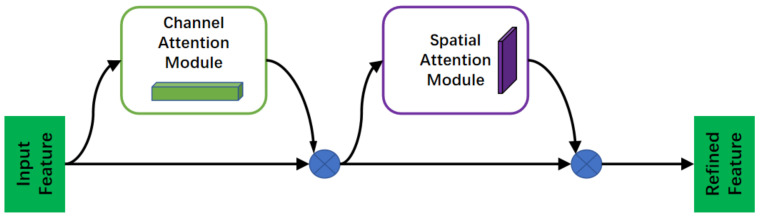
Convolutional Block Attention Module (CBAM).

Convolutional Block Attention Module (CBAM) [25] is a simple and efficient forward convolutional neural attention module. CBAM consists of two independent sub-modules: the channel attention module and the spatial attention module. The output of the convolutional layer first passes through the channel attention module to obtain a weighted result, which then goes through the spatial attention module for final weighting. This approach not only saves parameters and computational power but also ensures that it can be integrated as a plug-and-play module into existing network architectures. The diagram of the attention module is shown in Figure 3.

### 2.3. Discriminator and Loss Function

**Figure 4:**
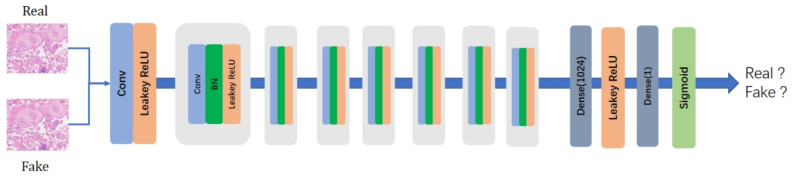
Architecture of our discriminator network.

In order to make the reconstructed image generate real high-frequency information, we add a feature discriminator under the original image discriminator [21]. The image discriminator, similar to the discriminators in traditional GAN models, takes the SR image as input to determine whether it is a real HR image or a SR image. The feature discriminator inputs the SR image into a VGG network to extract intermediate feature maps. Since the extracted feature maps contain structural information, the feature discriminator distinguishes between SR images and real HR images based not only on high-frequency components but also on structural components. Through adversarial training with the generator and the two discriminators, our generator is trained to synthesize realistic structural features rather than random high-frequency noise. The structure of our discriminator is shown in Figure 4.

Image discriminator $D^{i}$ and feature discriminator $D^{f}$ are next described. The image discriminator $D^{i}$ judges real HR images and SR images based on pixel values, while the feature discriminator $D^{f}$ distinguishes between real HR images and SR images through the mapping of feature maps. The generator loss function is as follows:

(8) $$C_{g}=C_{p}+\lambda \left(C_{a}^{i}+C_{a}^{f}\right)$$

$C_{p}$ is the perceptual loss [20]. $C_{a}^{i}$ is the generator’s image GAN loss for synthesizing high-frequency details in the pixel domain. $C_{a}^{f}$ is the generator’s feature GAN loss for synthesizing structural details in the feature domain. λ is the weights of the GAN loss terms. To train the discriminators $D^{i}$ and $D^{f}$, we minimize the losses $C_{d}^{i}$ and $C_{d}^{f}$, which correspond to $C_{a}^{i}$ and $C_{a}^{f}$, respectively. The generator and the discriminator are trained by alternately minimizing $C_{g}$, $C_{d}^{i}$, and $C_{d}^{f}$. The image GAN loss $C_{a}^{i}$ and image discriminator loss $C_{d}^{i}$ of the generator are defined as follows:

(9) $$C_{a}^{i}=-\log\left(D^{i}\left(I^{g}\right)\right)$$

(10) $$C_{d}^{i}=-\log\left(D^{i}\left(I^{h}\right)\right)-\log\left(1-D^{i}\left(I^{g}\right)\right)$$

where $D^{i}\left(I\right)$ is the output of the image discriminator. The feature GAN loss $C_{a}^{f}$ and feature discriminator loss $L_{d}^{f}$ of the generator are defined as follows:

(11) $$C_{a}^{f}=-\log\left(D^{f}\left(\varpi^{k}\left(I^{g}\right)\right)\right)$$

(12) $$C_{d}^{f}=-\log\left(D^{f}\left(\varpi^{k}\left(I^{h}\right)\right)\right)-\log\left(1-D^{f}\left(\varpi^{k}\left(I^{g}\right)\right)\right)$$

where $D^{f}\left(\varpi^{k}\right)$ is the output of the feature discriminator. During training, the generator can be made to produce realistic structural high-frequency details instead of noise artifacts.

## 3. Experimental Results and Discussion

### 3.1. Experimental Settings

We used cell images obtained from a 40× microscope as HR images and downsampled them using BD to obtain the corresponding LR images. Each of the four cells has 800 pairs of training datasets and 5 test datasets. The test datasets are each named Cell A, Cell B, Cell C, and Cell D. We use the Peak Signal-to-Noise Ratio (PSNR), Structure Similarity Index Measure (SSIM), and Learned Perceptual Image Patch Similarity (LPIPS) to verify the authenticity of the reconstructed images and detect image distortion in various models. Calculating PSNR and SSIM in the YCbCr color space reduces the influence of chroma on the results and aligns better with human visual perception. We also calculated the number of parameters for each model and the reconstruction time for a single cell image.

During model training, the input HR images are randomly cropped to a size of 192 × 192, and the quantity is increased by random horizontal flips, and $90^{\circ }$, $180^{\circ }$, and $270^{\circ }$ rotations. The LR images are obtained by downsampling the corresponding HR images by ×2 using bicubic downsampling, resulting in a size of 96 × 96. We set the weight λ in Equation (8) as $10^{-3}$. For $\Phi^{m}$ in Equations (11) and (12), we use the Conv5 layer of the VGG-19. We train our model using the ADAM optimizer with $\beta_{1}=0.9$ and $\beta_{2}=0.999$. The learning rate is initialized to $2\times 10^{-4}$ and multiplied by 0.5 after the 200-th epoch. Our model is implemented using the PyTorch framework and trained on an NVIDIA 3090 GPU, and training takes approximately 8 to 9 h to complete.

### 3.2. Comparison with Classic Models

We conducted ablation experiments on our proposed model. We trained it using the collected microscope image dataset and evaluated it on the Cell A dataset. As can be seen from the results in Table 1, the CBAM, RDB, and feature discriminator that we have added to the model have a better PSNR value and SSIM value.

Then, we compare our model with six classic image SR models, including EDSR, Enhanced Super-Resolution Generative Adversarial Network (ESRGAN) [26], very deep Residual Channel Attention Network (RCAN) [27], RDN, SRResnet [17] (less a discriminator compared to SRGAN), and RFGANM. And, from Table 2, it can be seen that our model achieves the highest PSNR and SSIM for reconstructing images. This is because we added RDAB in the generator of our model, allowing it to fully utilize the hierarchical features of the original LR image and focus the reconstruction on the main information of the feature maps. To avoid generating artifacts during reconstruction, we added a feature discriminator, which enables the generator to produce high-frequency features relevant to the HR image, and we made corresponding improvements to the model’s loss function. On the LPIPS metric, the best model is ESRGAN, with our model being a close second. Regarding model parameters, the best model is SRResnet, and our model differs by only 5.91 M. For single image reconstruction time, EDSR is the fastest, and our model is only 0.51 s slower. In summary, compared to other SR models, our model achieves the highest PSNR and SSIM, and good LPIPS, model parameters, and reconstruction times.

**Figure 5:**
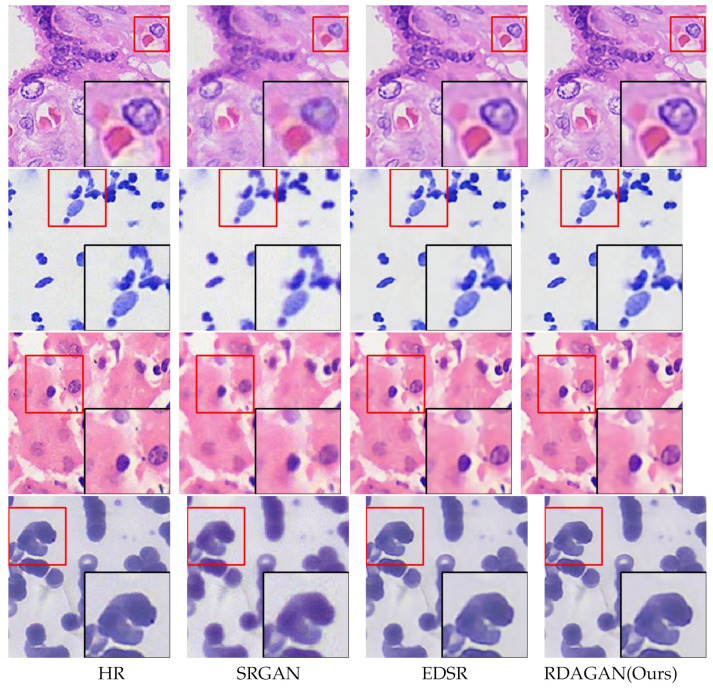
Comparison of reconstructed detail views for Cell A, Cell B, Cell C, and Cell D using our proposed model and two classical models.

In addition, Figure 5 shows the reconstruction detail views of our proposed model and two classic models. It can be observed that the cell detail textures and edge parts reconstructed by our model are closer to the HR images. In summary, these results demonstrate that our network model has achieved excellent performance.

## 4. Conclusions

In this paper, we provide a microscopic images dataset. And the SRGAN model is improved by modifying the residual block of the generator to an RDB and combining the attention mechanism. Additionally, a feature discriminator is introduced to ensure that the reconstruction avoids generating artifacts and instead produces high-frequency features pertinent to the image. The loss function is also enhanced. Finally, the experimental results demonstrate that our model outperforms the classical models and is hopeful to meet the demand for high-resolution imaging of large volume samples.

## Figures and Tables

**Table 1:** The ablation experiments results (“✓” denotes the corresponding operation).

	CBAM	RDB	Feature Discriminator	PSNR/dB ↑	SSIM ↑
1				28.12	0.72
2	✓			28.75	0.75
3	✓	✓		32.83	0.85
4	✓	✓	✓	33.13	0.87

**Table 2:** Comparison of PSNR, SSIM, LPIPS, model parameters, and reconstruction times for Cells A, B, C, and D using our proposed model and six classic image SR models (the red indicates the best result).

Model	Dataset	PSNR/dB ↑	SSIM ↑	LPIPS ↑	Parameters/M	Time/s
RFGANM [20]	Cell A	28.12	0.72	0.2048	5.92	2.27
	Cell B	27.45	0.85	0.1108		
	Cell C	28.86	0.77	0.1645		
	Cell D	29.62	0.84	0.1513		
ESRGAN [26]	Cell A	28.74	0.75	0.1089	102.16	2.70
	Cell B	31.71	0.90	0.0292		
	Cell C	30.49	0.79	0.0775		
	Cell D	33.50	0.89	0.0414		
SRResnet [17]	Cell A	30.98	0.82	0.1616	5.34	2.33
	Cell B	33.53	0.93	0.0385		
	Cell C	32.62	0.86	0.1356		
	Cell D	35.92	0.93	0.0572		
RCAN [27]	Cell A	31.06	0.82	0.1689	58.92	2.17
	Cell B	33.54	0.93	0.0412		
	Cell C	32.66	0.86	0.1466		
	Cell D	35.68	0.93	0.0590		
EDSR [18]	Cell A	31.63	0.84	0.1542	155.30	2.05
	Cell B	34.07	0.94	0.0362		
	Cell C	32.73	0.86	0.1414		
	Cell D	36.45	0.93	0.0606		
RDN [19]	Cell A	31.68	0.84	0.1531	50.14	3.23
	Cell B	34.13	0.94	0.0365		
	Cell C	32.81	0.86	0.1401		
	Cell D	36.89	0.94	0.0609		
RDAGAN	Cell A	33.13	0.87	0.1364	11.25	2.56
	Cell B	36.09	0.96	0.0259		
	Cell C	34.29	0.89	0.1272		
	Cell D	38.31	0.96	0.0550		

## Data Availability

The original contributions presented in the study are included in the article, further inquiries can be directed to the corresponding author/s.

## Abbreviations

The following abbreviations are used in this manuscript:

FOV	Field of View
SR	Super-Resolution
LR	Low-Resolution
HR	High-Resolution
SISR	Single Image Super-Resolution Reconstruction
RDAGAN	Residual Dense Attention Generative Adversarial Network
CNN	Convolutional Neural Network
VDSR	Very Deep Super Resolution
LapSRN	Laplacian Pyramid Super-Resolution Network
GAN	Generative Adversarial Network
SRGAN	Super-Resolution Generative Adversarial Network
EDSR	Enhanced Deep Super Resolution
RDB	Residual Dense Block
RDN	Residual Dense Network
RF-GANM	Registration-Free GAN
DCFA	Deep Fourier Channel Attention
RDAB	Residual Dense Attention Block
BD	Bicubic Downsampling
DFF	Dense Feature Fusion
ReLU	Rectified Linear Unit
GFF	Global Feature Fusion
GRL	Global Residual Learning
LFF	Local Feature Fusion
LRL	Local Residual Learning
CBAM	Convolutional Block Attention Module
CM	Continuous Memory
PSNR	Peak Signal-to-Noise Ratio
SSIM	Structural SIMilarity index
LPIPS	Learned Perceptual Image Patch Similarity
ESRGAN	Enhanced Super Resolution Generative Adversarial Network
RCAN	Residual Channel Attention Network

## Appendix A

### Appendix A.1

Table A1 introduces the image characteristics of the four microscopic cell datasets we collected using the MoticEasyScan NFC 300 microscope. We selected representative cells with distinctive features. The table provides detailed descriptions of the colors and structures within the cells. We hope this will help you understand the features of the training datasets.

**Table A1:** The characteristics of the four types of cells: Cell A, Cell B, Cell C, and Cell D.

	Dataset	Image	Image Feature
1	Cell A		The dark purple circular or oval structures in the image represent cell nuclei. The areas surrounding the nuclei are light pink, indicating the cytoplasm and extracellular matrix. There are also some small red circular structures.
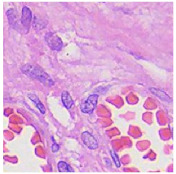
2	Cell B		The small dark blue circular or oval structures in the image represent cell nuclei. The cytoplasmic regions appear lighter blue or unstained.
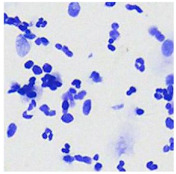
3	Cell C		The dark purple circular structures in the image represent cell nuclei. The areas surrounding the nuclei are light pink, indicating the cytoplasm.
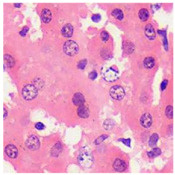
4	Cell D		The small dark blue circular or oval particles in the image are arranged in chains or clusters.
